# A review of breast cancer awareness among women in India: Cancer literate or awareness deficit?

**DOI:** 10.1016/j.ejca.2015.07.008

**Published:** 2015-09

**Authors:** A. Gupta, K. Shridhar, P.K. Dhillon

**Affiliations:** a South Asia Network for Chronic Disease (SANCD), Public Health Foundation of India, 4th Floor, Plot No. 47, Sector 44, Gurgaon 122002, India; b Centre for Chronic Conditions & Injuries, Public Health Foundation of India, 4th Floor, Plot No. 47, Sector 44, Gurgaon 122002, India

**Keywords:** Breast cancer, Awareness, Risk factors, Women, India

## Abstract

**Background:**

Breast cancer is the most common female cancer worldwide including India, where advanced stages at diagnosis, and rising incidence and mortality rates, make it essential to understand cancer literacy in women. We conducted a literature review to evaluate the awareness levels of risk factors for breast cancer among Indian women and health professionals.

**Methods:**

A structured literature search using combined keywords was undertaken on bibliographic databases including MEDLINE, Cochrane Database of Systematic Reviews, Cumulative Index to Nursing and Allied Health (CINAHL) and SCOPUS. Searches were restricted to research published in English language peer-reviewed journals through December, 2014 in India.

**Results:**

A total of 7066 women aged 15–70 years showed varied levels of awareness on risk factors such as family history (13–58%), reproductive history (1–88%) and obesity (11–51%). Literacy levels on risk factors did not improve over the 8-year period (2005–2013). On average, nurses reported higher, though still varied, awareness levels for risk factors such as family history (40.8–98%), reproductive history (21–90%) and obesity (34–6%). Awareness levels were not consistently higher for the stronger determinants of risk.

**Conclusion:**

Our review revealed low cancer literacy of breast cancer risk factors among Indian women, irrespective of their socio-economic and educational background. There is an urgent need for nation- and state-wide awareness programmes, engaging multiple stakeholders of society and the health system, to help improve cancer literacy in India.

## 1 Background

Breast cancer is the most common female cancer worldwide representing nearly a quarter (23%) of all cancers in women [1,2]. The global burden of breast cancer is expected to cross 2 million by the year 2030, with growing proportions from developing countries [3]. Although age-standardised incidence rates in India are lower than in the United Kingdom (UK) (25.8 versus 95 per 100,000), mortality rates are nearly as high (12.7 versus 17.1 per 100,000, respectively) as those of the UK [1]. Breast cancer incidence rates within India display a 3–4-fold variation across the country, with the highest rates observed in the Northeast and in major metropolitan cities such as Mumbai and New Delhi [4]. Reasons for this variation include differences in demographic (e.g., education), reproductive (e.g., age at first child and number of children), anthropometric (e.g., adiposity) and lifestyle factors (e.g., tobacco smoking and alcohol use).

Diagnosis at advanced stages of disease contributes to the high mortality rate among women due to breast cancer, which can be attributed to low levels of awareness, cumbersome referral pathways to diagnosis, limited access to effective treatment at regional cancer centres and incomplete treatment regimens [3,5–10]. With the rising breast cancer incidence in India [4] and disproportionately higher mortality [11], it is essential to understand the level of cancer literacy, especially since the average age at diagnosis is 10 years younger than women in Western countries [12]. An assessment of existing levels of cancer awareness is a pre-requisite for planning comprehensive health programmes, early detection and treatment campaigns [13], that effectively engage communities of women and men.

Despite long-standing national programmes, such as the National Cancer Control Programme launched in 1975, under the National Programme for cardiovascular disease, diabetes, cancer and stroke (NPCDCS launched under the 12th five year Plan from 2012 to 2017) [14], to increase awareness and early detection behaviours, the mortality rates for breast cancer continue to rank the highest in the country [11]. Barriers such as ‘low cancer awareness’, also referred to as ‘awareness deficit’ or ‘scarcity of awareness’ among women, the presence of stigma, fear, gender inequity and reduced engagement in screening behaviours, such as breast self-examinations, contribute to high mortality rates [8]. We conducted this review to evaluate cancer literacy in Indian women, of breast cancer risk factors, which include age, family history, age at first birth, parity, duration of breastfeeding, adiposity and alcohol use (Table 1) [15,16].

## 2 Materials and methods

The methodology of the Cochrane Handbook for Systematic Reviews [17] was followed through a search of several electronic databases including MEDLINE, Cochrane Database of Systematic Reviews, Cumulative Index to Nursing and Allied Health (CINAHL) and SCOPUS. Searches were restricted to research published in the English language peer-reviewed journals, as well as grey literature, till December 2014. From these publications, the bibliographic lists were also hand-searched for additional papers.

Index terms (MeSH terms) used for the search were, ((breast neoplasms OR breast cancer OR breast health) AND (awareness OR knowledge OR attitude OR education^∗^ OR programme) AND (women OR female OR health worker^∗^ OR health professional^∗^) AND (risk factor^∗^ OR risk assessment) AND India). We also searched for qualitative studies on breast cancer using the above mentioned search terms. No qualitative study was found on breast cancer awareness among women in India. The initial search yielded 120 studies on the basis of terms in the titles and abstracts (where available) identified from the search strategy. Studies that focused on awareness of screening, or treatment modalities alone for breast cancer were excluded as we focused exclusively on the literacy levels of risk factors and causes of breast cancer. After applying the inclusion criteria, full-text articles were retrieved for 20 studies, of which 13 fulfilled the eligibility.

We considered risk factors (Table 1) summarised in a systematic review conducted by an expert panel committee of the International Agency for Research on Cancer (IARC), the World Cancer Research Fund (WCRF) and the American Institute of Cancer Research (AICR). They classified breast cancer risk factors on the basis of the strength of existing evidence such as sufficient/convincing evidence; insufficient/weak evidence and no conclusive evidence [15,16].

## 3 Results

**Fig. 1:**
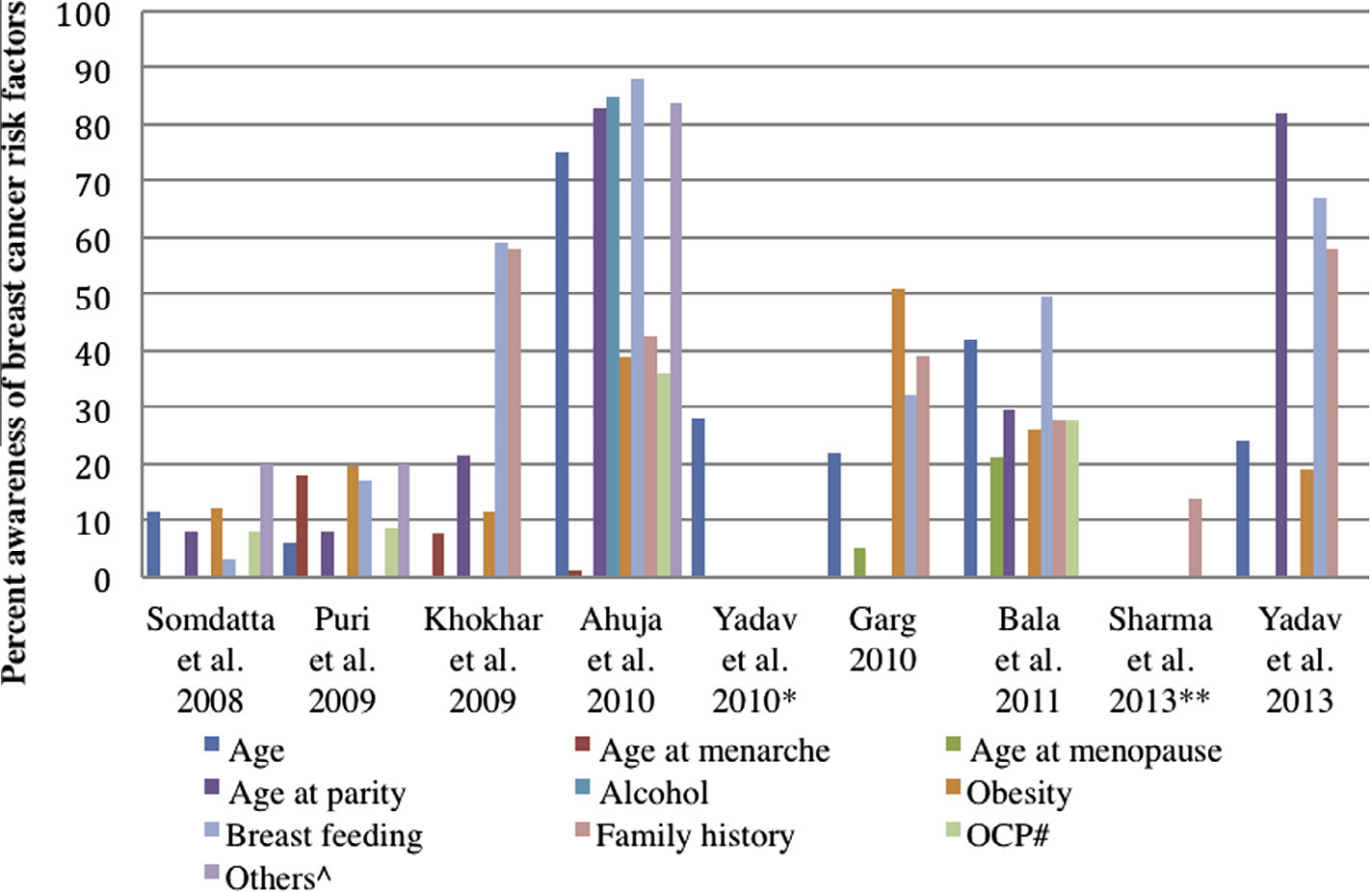
Percent awareness of breast cancer risk factors from studies^@^ in the general female population of India; ^@^The study by Rao et al. [20] is not shown, as there was no data on percent awareness of individual risk factors. Only overall awareness (9%) was reported. ^#^Oral Contraceptive Pills (OCP). ^Trauma/Stress/abortion/radiation/’lifestyle’/Hormone Replacement Therapy (HRT). ^∗^Yadav et al. (2010) [17] assessed knowledge of the breast cancer risk factor through only one question on ‘age’. The study broadly focussed on assessing the knowledge, symptoms, likelihood of developing breast cancer and awareness of diagnostic modalities for breast cancer. ^∗∗^Sharma et al. (2013) [19] evaluated knowledge of the breast cancer risk factor through only one question on ‘family history’. The study broadly focussed on assessing the awareness of common symptoms, methods of early detection and practice of breast self-examination (BSE) and clinical breast examination (CBE).

The literature search yielded 120 articles, of which 13 studies met the inclusion criteria for this study. These studies included information on 7066 women aged 15–70 years [18–30] (Table 2). Of these, 10 studies were conducted among women in community settings and three among health professionals, comprising nurses and nursing students [24,26,29]. Studies in the general population (*n* = 10) included adult women with a mean age ranging from 25 to 48 years. While the majority of studies were conducted in urban cities such as Ahmedabad, Mumbai, Jaipur and Chandigarh; and states such as Haryana and New Delhi [18,19,23,25,27,30], several were conducted in rural settings (e.g., Haryana and coastal villages of Karnataka), peri-urban and slum settings (e.g., Chandigarh and Medchal Mandal village in Andhra Pradesh [20,31]), as well as the metropolitan city of Mumbai [21,28,30] The majority of studies consisted of married women, involved in household work (Table 2), with illiteracy rates ranging from 8% to 46%. Four studies [19,21,23,28] reported the socio-economic (SES) of participants measured according to the Kuppuswamy socio-economic scale [32], which is based on monthly family income (Rao et al., 2005 – 58% lower class; Somdatta, 2008 - 34% upper lower class; Khokhar, 2009 – 58% upper middle class and Ahula et al., 2009 – 48% lower class). Literacy levels of known risk factors among women such as age, family history, number of children, age at menarche, age at menopause and age at birth of first child varied widely (Fig. 1). Awareness levels on the strongest risk factors related to age at menarche and age at menopause varied from 1% to 21% [27,28] while 13–58% reported family history as a risk factor for breast cancer [20,27]. Age at birth of first child and that of breast feeding were considered to be risk factors by 8–83% and 17–88% of the women, respectively [22,28]. Tobacco smoking was reported to be a risk factor in 20–74% of women [19,28]. There were no studies reporting literacy levels on number of children as a risk factor for breast cancer. Obesity and overweight were considered to be risk factors by 11–51% [19,25].

**Fig. 2:**
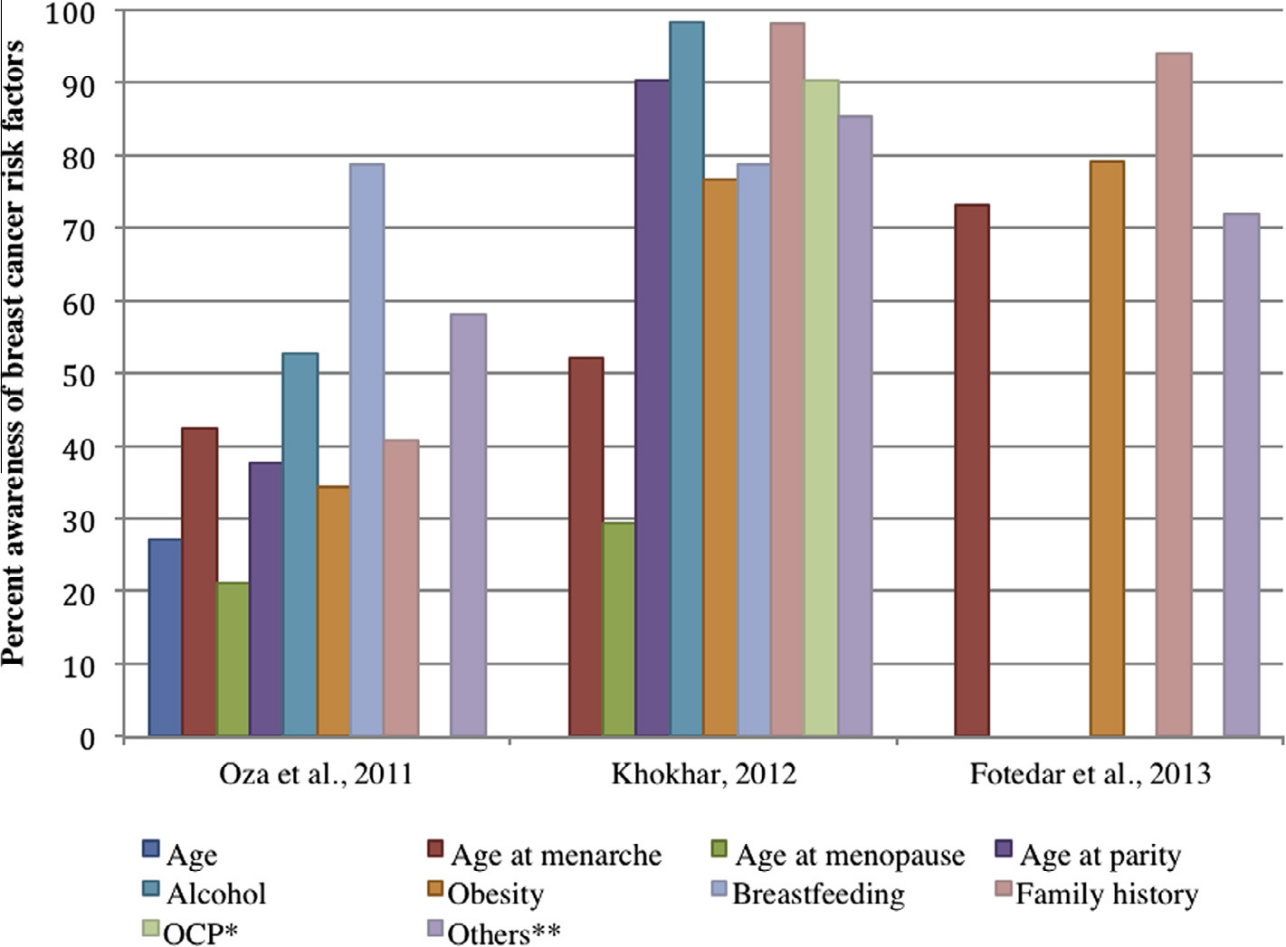
Percent awareness of breast cancer risk factors from studies among health professionals (nurses and nursing students) in India. ^∗^Oral Contraceptive Pills (OCP). ^∗∗^Trauma/Stress/abortion/radiation/’lifestyle’/Hormone Replacement Therapy (HRT).

Studies among health professionals comprised of nurses and nursing students (*n* = 3), included adult women with a mean age ranging from 28 to 40 years. All three studies were conducted in urban settings (Ahmedabad, Shimla and New Delhi), with educated women, either studying or practicing nursing (Table 2). One of the studies on nurses reported high literacy levels for risk factors such as family history (98%) and age at first child (90%) [24] (Fig. 2). Of these studies, 52–98% of nurses and nursing students reported smoking and alcohol as risk factors. Literacy levels on family history and reproductive history were similar in nurses, but varied across study populations, ranging from 40% to 95% (Fig 2). Only one study was found to have reported 42% of literacy levels on number of children as a risk factor for breast cancer [29]. The percent awareness of risk factors, such as age at birth of first child, age at menarche, family history, alcohol consumption and obesity was observed to have increased with each successive year for the studies conducted over the 4-year period (2008–2012), indicating a possible increase in cancer literacy in this group (Fig. 2).

## 4 Discussion

A review of the literature reveals low breast cancer literacy with regard to risk factors among Indian women, irrespective of their socio-economic and educational backgrounds, with little correlation between awareness levels and strength of evidence of the risk factors. When plotting the studies in chronological order (Fig. 1), we found no increase in the cancer literacy over time; low levels of awareness were consistently observed for important risk factors such as age at menarche, age at menopause and age at birth of first child in the general population. This may not be true for nurses/nursing students, in whom improved literacy of risk factors was observed in more recent studies. In general, we found relatively low, and wide, variation in awareness of risk factors for breast cancer among women in India over the 8-year period of publications, even as breast cancer became the most common cancer in the country. Women more commonly believed that unhealthy habits related to alcohol and tobacco consumption were more important risk factors than reproductive history, which is a much stronger determinant of breast cancer [33–36]. Long-term data on fertility patterns [37,38] show on average, Indian women are having fewer children (average of 3.39 live born children in 1992–1999 and 2.68 in 2005–2006) and marrying at older ages (% married by the age of 18 years has declined from 54.2% in 1992–1993 to 44.5% in 2005–2006), though it is difficult to extrapolate the impact of such changes over time in terms of excess risk [39] due to limited data.

Nurses were more likely to report on risk factors with less consistent evidence (Table 1), such as dietary factors, exposure to ionising radiation and tobacco consumption, when compared to risk factors such as family history and age at menarche, which are consistent, known risk factors for breast cancer [15,16]. A better understanding of risk factors such as age at birth of first child and alcohol consumption over the four-year period during which these studies were conducted was observed in nurses and/or nursing students, indicating a potential increase in literacy level among health professionals. However, a short time span and small sample size precluded a time trends evaluation, and populations may not be directly comparable on important characteristics influencing literacy. Literacy deficit among health professionals is recognised as a potential barrier in breast cancer prevention and early detection, given their leading role and contribution in spreading awareness, particularly in primary care settings across the globe [40–42]. Our study also reveals that health professionals’ (i.e. nurses and nursing students) awareness on the strength of risk factors for breast cancer was limited for guiding the patients towards important modifiable means of prevention.

There is an urgent need to explore the drivers of awareness deficits and stigma surrounding breast cancer, both in the general population and among health care professionals, as incidence and mortality rates continue to rise [43]. Understanding the drivers and barriers is important for strategic and effective awareness campaigns and/or interventions on prevention and early detection. A recent systematic review of educational interventions in improving subjective cancer risk perception in 40 studies (*n* = 12 randomised controlled trials (RCTs) and *n* = 28 prospective observational studies; *n* = 29/40 on breast cancer) from high income countries did not show a significant change in literacy level after educational interventions on risk perception from baseline [44]. A separate analysis of prospective observational studies in the same systematic review however, yielded significant changes in the level of perceived risk and improved risk accuracy among cancer patients [44]. The applicability of these results in Indian settings is uncertain. Similar reviews on educational interventions in Low Middle Income Countries (LMIC) settings are required, to understand the cultural and socio-economic context, including important factors such as stigma, for influencing awareness levels. A consensus review from the Breast Health Global Initiative (BHGI) 2010 Global Summit summarising barriers to breast cancer care, highlighted the lack of or very limited access to treatment, and limited knowledge of health professionals as major barriers to cancer prevention and detection in developing countries [45–47]. Low awareness levels of risk factors are also a consequence of low informed coverage through different forms of media, including television and newspaper [31,48,49]. In India, the media publicity and policy efforts on cancer have primarily focused on the reduction and perils of tobacco use [50]; there has been little discussion of other important risk factors such as alcohol, reproductive history and overweight/adiposity for example [51].

This review has certain limitations. These findings may not be generalised across the country as they are derived from 9 locations in northern, western, and southern geographical regions of the country. India’s heterogeneous populations with different socio-economic, cultural and social factors may yield wider variations than observed, though it is unlikely that literacy levels would be higher as this review includes major metropolitan cities such as Mumbai. The number of studies and time span are too narrow for an evaluation of significant changes in cancer literacy over time. While we emphasise the importance of literacy in this review, higher awareness levels in Indian women may not always translate to better outcomes. Some evidence suggests a noncompliant attitude of women after breast cancer screening in terms of diagnostic follow up for abnormal findings, irrespective of their socio-economic status, suggesting other factors related to fear, stigma, discrimination, denial, and distrust in the health system [52–59].

## 5 Conclusion

Indian women need to be aware of both modifiable and non-modifiable risk factors for breast cancer to adopt appropriate practices for prevention. There is an urgent call for more effective nation- and state-wide cancer literacy programmes, as well as engagements with community-level organisations and the health system [60]. With wide variations in the state-level burden, a coordinated, intensive health promotion intervention programme on risk factors, prevention, screening and management for breast cancer is prudent. Training on the latest evidence regarding breast cancer risk factors should be offered to healthcare providers and community workers to raise their cancer literacy so they can then transmit this knowledge to other sections of the society. Continuing medical education programmes with enhanced emphasis on breast cancer in the curricula of nursing at institutional level and other healthcare training institutions should be a priority for women’s health in the country.

## Ethical approval

Not required.

## Role of funding source

Not involved in data collection, analysis and interpretation of findings.

## Source of funding

Wellcome Trust Strategic Award; South Asia Network for Chronic Disease (WT 084674).

## Conflict of interest statement

None declared.

## Figures and Tables

**Table 1:** IARC and WCRF/AICR evaluations of ‘modifiable’⁎risk factors for breast cancer in women [15,16].

Sufficient/convincing evidence	Insufficient/weak evidence	No conclusive evidence
*Increase risk*	*Increase risk*	
Alcohol consumption	Total dietary fat	Meat
Body fatness (post-menopausal)	Greater birth weight (pre-menopausal)	Fish
Adult height (post-menopausal)	Tobacco smoking	Folate
Any use of oral contraceptive pills (OCP)	Hormone replacement therapy	Vitamin D
Age at first child birth		Calcium
		Selenium
*Decrease risk*	*Decrease risk*	Dietary fibre
		Glycemic index
Lactation	Fruits and vegetables	Soya based foods
Body fatness (pre-menopausal)	Physical activity	Total energy intake
		Milk and dairy products

⁎ Apart from modifiable (or preventable) risk factors, there are several non-modifiable factors with convincing evidence such as age, sex, family history, high-risk genes, benign breast conditions, high oestrogen levels (e.g., early menarche and late menopause) and mammographic density [61].

**Table 2:** Studies on breast cancer awareness in general female population and among nurses/nursing students in India.

Study	Study design, sample size	Location	Population characteristics	Awareness level (%) of risk factors
*General population*				
Rao et al. (2005)	Community-based educational intervention study (*N* = 342)	Coastal Villages in Southern India	Rural population Age: 30–39 years (55.6%) Education: 18.1% illiterate Occupation: 63.5% housewife Marital status: 86.3% married Religion: 84% Hindu SES: 58.2% Lower class	Any risk factors 9%
Somdatta et al. (2008)	Cross-sectional study (*N* = 358)	New Delhi	Resettlement colony Mean age: 36 years Education: 46% illiterate Marital status- (not specified) SES: 34% Upper-lower class	Increasing age 4.9% Obesity 11% Breast feeding 3% Oral Contraception Pills (OCP) 8% Tobacco use 20% Trauma 20%
Puri et al. (2009)	Cross-sectional study (*N* = 981)	Chandigarh	Peri-urban and slum population Mean age: 29.1 years Education: 17.4% illiterate Occupation: 83.3% housewife Marital status: 87.1% married SES: (not specified)	Age at marriage 5.9% Age at menarche 17.8% Obesity 19.5% Breast feeding 16.9% OCP 8.6% Abortion 11.6% Exposure to radiation 8.9%
Khokhar (2009)	Cross-sectional study (*N* = 441)	New Delhi	Urban population Mean age: 37.2 years Education: No illiterate Occupation: 100% Teaching Marital status: 80.7% married Religion: 83.7% Hindu SES: 58.3% Upper middle class	Age at first child 21.3% Age at menarche 7.7% Obesity 11.6% Breast feeding 59.2% Family history 58.0%
Ahuja et al. (2010)	Cross sectional study (*N* = 80)	Mumbai	Rural population Mean age: 48.3 years Education: 38.8% illiterate Occupation: 60% housewife Marital status: 100% married SES: 48.8% Lower class	Age 81.3% Age at first child 83% Age at menarche 1% Alcohol 85% Obesity 38.8% Breast feeding 88% Family history 42.5% OCP 36% Stress 83.8% Smoking 74% Sedentary life style 15%
Yadav and Jaroli (2010)	Cross sectional study (*N* = 407)	Jaipur	Urban population Median age: 21 years Education: college going Marital status: 100% Unmarried SES: (not specified)	Age 28%
Garg (2010)	Cross sectional study (*N* = 970)	Chandigarh	Urban population Mean age: 26.2 years Education: 67% college going Marital status: (not specified) SES: (not specified)	Age 22% Age at menopause 5% Obesity 51% Breast feeding 32% Family history 39%
Bala and Gameti (2011)	Educational intervention study (*N* = 250)	Ahmedabad	Urban population Mean age: 33.7 years Education: 8% illiterate Occupation: 52.4% housewife Marital status: 90.8% married SES: (not specified)	Increasing age 42% Age at first child 29.6% Age at menopause 21.2% Obesity 26% Breastfeeding 49.6% Family history 27.6% OCP 27.6%
Sharma et al. (2013)	Cross-sectional study (*N* = 300)	Medchal Mandal village, Ranga Reddy District, Andhra Pradesh	Peri-urban and rural population Mean age: 26.5 years Education: 28.6% illiterate Marital status: 88.3% married SES: (not specified)	Family history 13.7%
Yadav et al. (2013)	Cross-sectional study (*N* = 300)	Haryana	University, urban and rural population Mean age: 25 years (University); 30 years (urban); 39 years (rural) Education: (not specified) Marital status: (not specified) SES: (not specified)	Age (61% rural; 24% urban; 50% university) ‘Late pregnancy’ (older than 30 years of age) (5% rural; 82% urban; 50% university) Overweight (19% rural; 19% urban; 53% university) Breastfeeding (69% rural; 67% urban; 63% university) Family history (31% rural; 58% urban; 67% university)

*Health professionals (nurses/nursing students)*				
Oza et al. (2011)	Cross sectional study (*N* = 250)	Ahmedabad	Urban population Mean age: 40.6 years Education: Nurses Marital status: (not specified) SES: (not specified)	Age at first child 37.6% Age at menopause 27.2% Age 27.2% Alcohol 52.8% Obesity 34.4% Breast feeding 78.8% Family history 40.8% Nulliparity 42.4% Dietary fat 20.4% Ionising radiation 58%
Khokhar (2012)	Cross sectional study (*N* = 259)	New Delhi	Urban population Mean age: 35.7 years Education: Nursing education Marital status: 81.4% married Religion: 74.9% Hindu SES: (not specified)	Age at first child 90.3% Age at menarche 52.1% Age at menopause 29.3% Alcohol 98.45% Obesity 76.4% Breast feeding 96.1% Family history 98.1% OCP 90.3% Hormone Replacement Therapy (HRT) 85.3%
Fotedar et al. (2013)	Cross-sectional study (*N* = 434)	Shimla	Urban population Mean age: 28 years Marital status: 45% married Education: Nursing education SES: (not specified)	Age at menarche 73.1% Family history 93.9% Diet 79.2% Ionising radiation 71.9%
